# Pyrazine and Phenazine Heterocycles: Platforms for Total Synthesis and Drug Discovery

**DOI:** 10.3390/molecules27031112

**Published:** 2022-02-07

**Authors:** Robert W. Huigens, Beau R. Brummel, Srinivasarao Tenneti, Aaron T. Garrison, Tao Xiao

**Affiliations:** Center for Natural Products, Drug Discovery and Development (CNPD3), Department of Medicinal Chemistry, University of Florida, 1345 Center Drive, Gainesville, FL 32610, USA; BBrummel@dental.ufl.edu (B.R.B.); stenneti83@gmail.com (S.T.); aaron.garrison@astrazeneca.com (A.T.G.); xtxiaotao1987@gmail.com (T.X.)

**Keywords:** pyrazine, phenazine, heterocyclic chemistry, total synthesis, medicinal chemistry

## Abstract

There are numerous pyrazine and phenazine compounds that demonstrate biological activities relevant to the treatment of disease. In this review, we discuss pyrazine and phenazine agents that have shown potential therapeutic value, including several clinically used agents. In addition, we cover some basic science related to pyrazine and phenazine heterocycles, which possess interesting reactivity profiles that have been on display in numerous cases of innovative total synthesis approaches, synthetic methodologies, drug discovery efforts, and medicinal chemistry programs. The majority of this review is focused on presenting instructive total synthesis and medicinal chemistry efforts of select pyrazine and phenazine compounds, and we believe these incredible heterocycles offer promise in medicine.

## 1. Introduction

**Figure 1:**
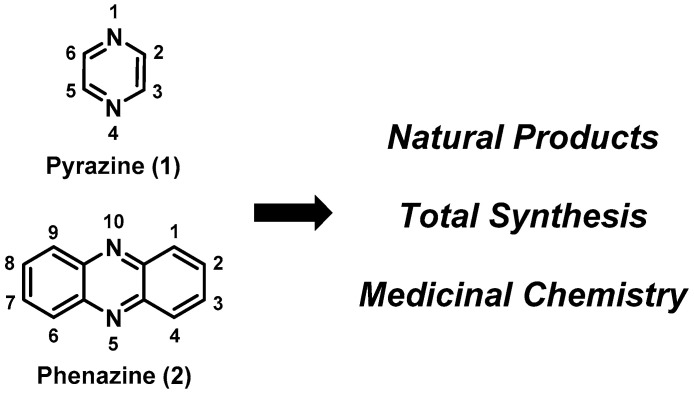
The chemical structures of pyrazine (**1**) and phenazine (**2**).

Pyrazine- and phenazine-containing small molecules and natural products collectively demonstrate a breadth of biological activities that are of significant interest to human health and medicine [1,2]. The pyrazine (**1**; Figure 1) heterocycle is composed of a six-membered aromatic structure bearing two nitrogen atoms, arranged in a 1,4-orientation embedded within a carbon framework [1,2,3]. Phenazine (**2**) [4,5] heterocycles contain fused benzene moieties at the carbon positions of a pyrazine nucleus. Both pyrazines and phenazines possess distinct chemical reactivity profiles, and have been the focal point of advances in total synthesis, synthetic methods, chemical biology, and drug discovery [1,2,3,4,5,6]. This review presents a sampling of total synthesis and medicinal chemistry campaigns of various pyrazine- and phenazine-containing compounds.

## 2. Pyrazines and Phenazines of Therapeutic Interest

There have been many biological investigations related to pyrazine- and phenazine-containing compounds that demonstrate therapeutic value related to human health and disease. The World Health Organization’s (WHO) Model List of Essential Medicines in 2019 included four pyrazine (amiloride, bortezomib, paritaprevir, pyrazinamide) and one phenazine (clofazimine) drug molecules [7]. The pyrazine scaffold offers additional promise, as computational studies have shown common drug–target interactions related to biologically active pyrazines, lending credence to incorporating this heterocycle, along with phenazine, into appropriate drug design campaigns [8]. 

### 2.1. Pyrazines

**Figure 2:**
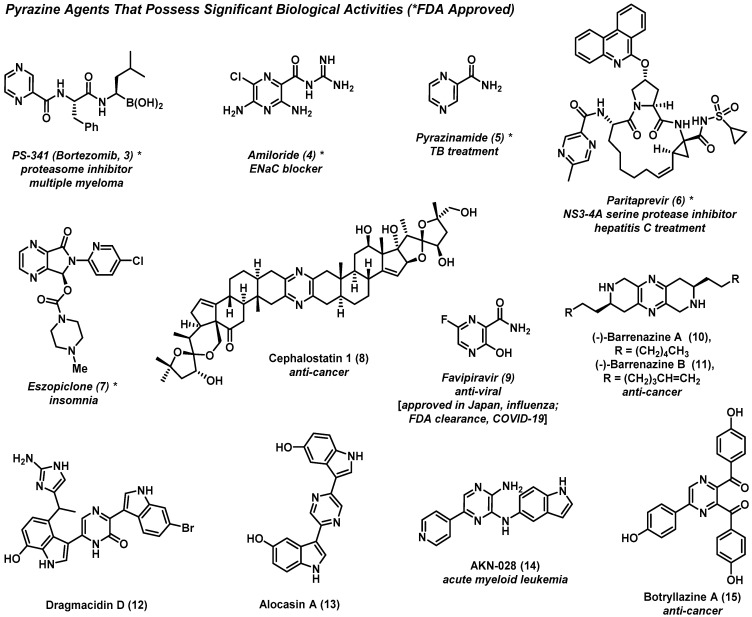
Pyrazine heterocycles that possess a diversity of biological activities, including multiple therapeutic agents used in human medicine.

In this section, we provide a brief overview of select pyrazine agents that demonstrate biological activity, including some therapeutic agents (see Figure 2). Additional discussions are included for select pyrazine compounds in later sections when discussing relevant total syntheses, synthetic methodological advances, or drug discovery campaigns.

Bortezomib (PS-341, **3**) is a first-in-class treatment for multiple myeloma, and was the first FDA-approved proteasome inhibitor to see clinical use [9,10]. This pyrazine-containing therapeutic agent reversibly inhibits the 26S proteasome through the action of its boronic acid moiety, leading to the dysregulation of proteins critical to multiple myeloma cell growth and survival. Bortezomib’s boronic acid is known to complex the key hydroxyl group of a threonine side chain in the proteasome’s active site to inhibit proteolysis.

Eszopiclone (**7**) is a pyrrolopyrazine-based sedative that has been widely used to treat insomnia [11,12] since receiving FDA approval in 2004. Interestingly, eszopiclone was declined approval in the EU owing to similarity to the racemate, which was patented. Eszopiclone is the (*S*)-enantiomer of the hypnotic agent zopiclone, whereas the (*R*)-enantiomer has greatly reduced sedative activities. Eszopiclone is believed to elicit its effects through modulating GABA_A_ receptor domains, which are related to benzodiazepine receptors.

Cephalostatin 1 (**8**) is a naturally occurring pyrazine isolated from the marine worm *Cephalodiscus gilchristi*, and demonstrates remarkably potent activities against multiple cancer types and cell lines [13]. Incredibly, cephalostatin 1 reported an average GI_50_ = 1.8 nM across all 60 cancer cell lines in the NCI-60 human cancer cell line panel. This complex natural product, along with the related ritterazine alkaloids, [14] contains a pyrazine nucleus flanked by stereochemically complex, oxygenated steroidal units containing spiroketal moieties. Interestingly, cephalostatin 1 demonstrates potential synthetic lethality with the *p16* tumor suppressor gene, which would allow this naturally occurring pyrazine to selectively kill cancer cells with altered or mutated *p16*. Total synthesis of cephalostatin 1 has been pursued by multiple labs in efforts to determine its cellular target and the therapeutic potential for this natural product to treat cancer, since only small quantities of **8** have been isolated from marine sources, which has hampered its investigation in clinical trials [15].

Favipiravir (**9**) is a pyrazine prodrug therapy that inhibits RNA-dependent RNA polymerase of the influenza virus [16]. This agent also demonstrates inhibitory action against several other pathogenic RNA viral infections (e.g., arenavirus, bunyavirus, norovirus), and has shown promising therapeutic potential against Ebola viral infections. Currently, favipiravir is being investigated for use against COVID-19 infection in several countries around the world, with some promising preliminary findings; however, additional studies are needed in order to determine its clinical potential [17,18].

The small molecule AKN-028 and the botryllazine natural products demonstrate a diversity of biological activities. AKN-028 (**14**) is a novel pyrazine-based tyrosine kinase inhibitor that has produced promising results in preclinical studies against acute myeloid leukemia (AML) [19,20]. Botryllazine A (**15**) is a pyrazine-containing natural product isolated from the tunicate *Botryllus leachii* [21]. In addition to the initially reported antineoplasmic activity, botryllazine B has demonstrated inhibitory activity against human aldose reductase (ALR2), which could be useful for attenuating diabetic complications [22].

### 2.2. Phenazines

**Figure 3:**
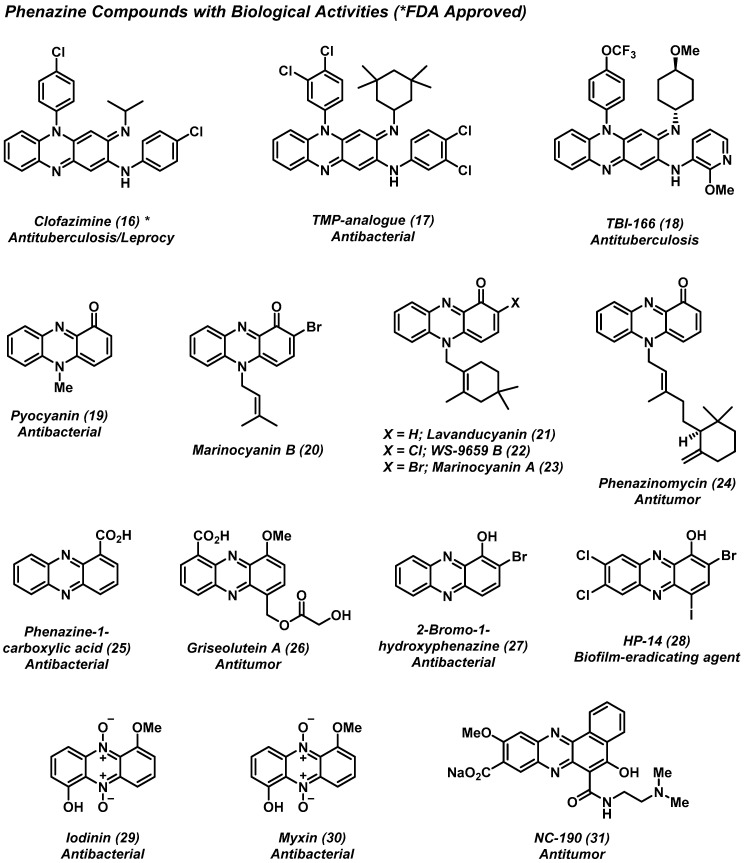
Phenazine heterocycles that demonstrate biological activities, including FDA-approved clofazimine.

The phenazine heterocycle is showcased in numerous biologically active natural products and synthetic small molecules. In this section, we provide a concise overview of select phenazine agents that demonstrate activities relevant to human health and disease. Phenazine natural products are produced by several bacteria, including pseudomonads, streptomycetes, and actinomycetes [4,5,23]. Collectively, natural and synthetic phenazines (Figure 3) display interesting anticancer, antimalarial, and antimicrobial activities [4,5,24,25,26,27,28,29]. Additional discussions are included for phenazines of interest in later sectiwhons of this review (e.g., lavanducyanin, marinocyanins, iodinin, myxin).

Clofazimine (**16**) is an antimycobacterial agent initially investigated as a treatment for *Mycobacterium tuberculosis* infections; however, this phenazine derivative later received FDA approval to treat lepromatous leprosy (caused by *Mycobacterium leprae*) in 1986 [28,29]. Clofazimine is often administered in combination with rifampin and/or dapsone due to the high frequency of resistance that many leprosy infections have to these antibiotics [30]. In addition, clofazimine is on the World Health Organization’s (WHO) model list of essential medicines, illustrating its global therapeutic impact [7].

Pyocyanin (**19**) and phenazine-1-carboxylic acid (**25**) are two phenazine antibiotics that are produced by *Pseudomonas aeruginosa*. Collectively, phenazine antibiotics are a colorful series of redox-active metabolites that endow their producing organism with various survival advantages. Phenazine antibiotics have been the source of inspiration for the discovery of halogenated phenazine compounds (e.g., HP-14 or **28**) that eradicate surface-attached bacterial biofilms, which will be discussed in a later section in this review.

Phenazinomycin (**24**) is a naturally occurring phenazine isolated from *Streptomyces* sp. WK-2057. This antibacterial antitumor agent bears a cyclic terpenoid appendage, and shows moderate antibacterial activities against *Staphylococcus aureus*. In addition, phenazinomycin demonstrated promising efficacy *in vivo* in a tumor model (with Sarcoma 180 cells) in mice, resulting in an increase in life span of up to 140% [31].

NC-190 (**31**) is a small benzo[*a*]phenazine molecule that demonstrates anticancer activity against multiple cancer cell lines *in vitro* and *in vivo* (tumor models in animals) [32,33]. NC-190, along with its methyl ester NC-182 (structure not shown), induces topoisomerase-II-dependent DNA cleavage and subsequent fragmentation [34,35]. NC-190 has demonstrated dose-dependent growth inhibition against HL-60 leukemic cells comparable to that of etoposide—a clinically used cancer therapy.

## 3. Select Total Syntheses of Natural Pyrazine Products

There are several pyrazine-containing natural products of interest due to their significant biological activities related to multiple disease states (e.g., cephalostatin 1, cancer; dragmacidin D, neurological diseases). The following section details select total syntheses of naturally occurring pyrazines showcasing a diversity of pyrazine reactivity and instructive synthetic routes.

### 3.1. Total Synthesis of Ritterazine B

Ritterazine B (**32**) is a bis-steroidal pyrazine (BSP) marine natural product isolated from the tunicate *Riterella tokioka* off Japan’s Izu Peninsula in 1995 [14]; it has gained a significant amount of interest due to it being considered “among the most potent growth inhibitors ever tested” by the National Cancer Institute (NCI) [36,37]. Ritterazine B possesses subnanomolar activity against P388 leukemia cells (IC_50_ = 0.17 nM) [38] and an average GI_50_ of 3.2 nM in the NCI-60 human cancer cell line panel [37,38,39]. BSPs are known to induce apoptosis in cancer cells; however, a COMPARE analysis suggests that these natural products act via a novel mode of action when compared to traditional cancer therapies [40]. Recent studies have revealed BSPs to be high-affinity ligands for oxysterol-binding proteins, while additional studies indicate that the endoplasmic-reticulum-specific heat-shock protein GRP78 could be their molecular target [41,42,43]. Unfortunately, these promising foundational studies have been hampered due to the lack of natural materials; thus, synthesis of **32** is required in order to fully evaluate its potential as a cancer therapy.

**Scheme 1:**
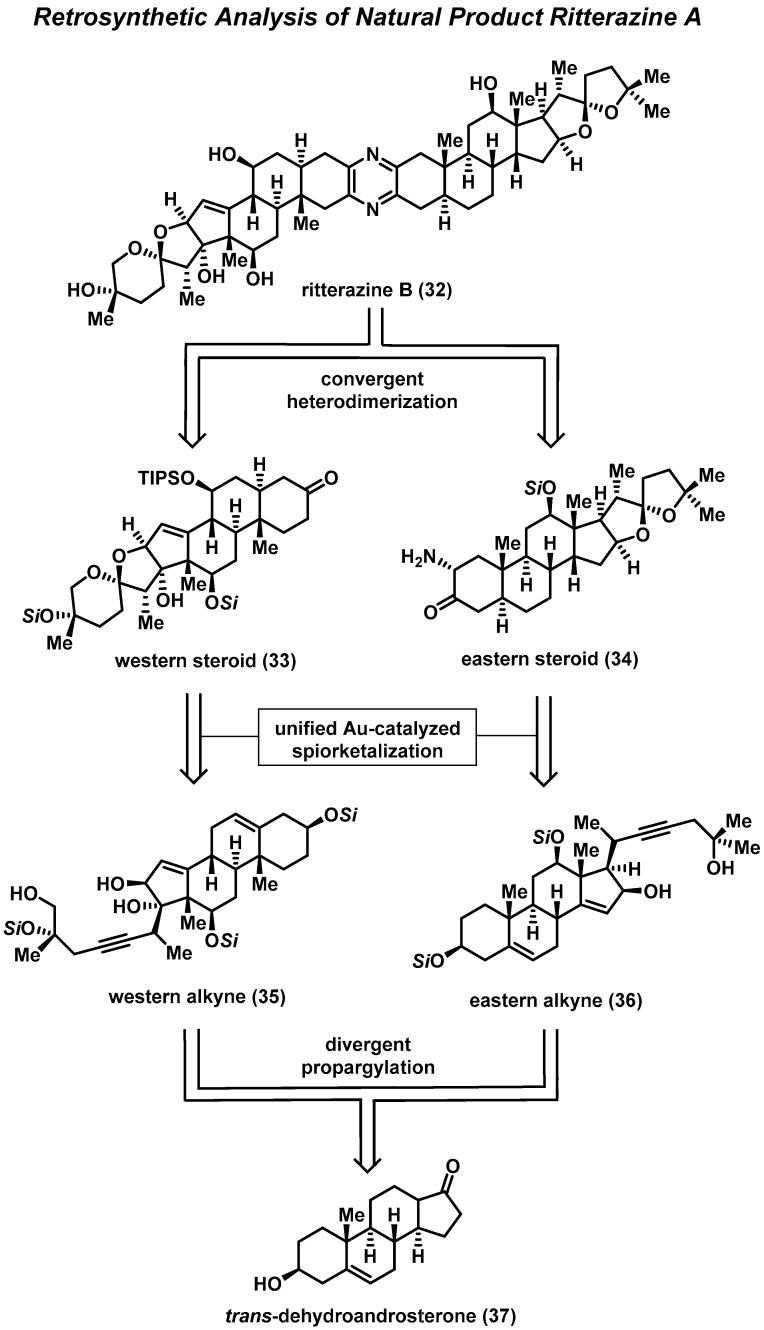
Reisman’s retrosynthetic analysis of ritterazine B (**32**) from *trans*-dehydroandrosterone (**37**).

In 2021, the Reisman Group reported the first total synthesis of ritterazine B (**32**), which featured a titanium-mediated propargylation, gold-catalyzed spirocyclization, and late-stage chromium C-H oxidation (Scheme 1) [44]. Disconnection of the central pyrazine ring of ritterazine B provided western and eastern fragments—steroids **33** and **34**, respectively. Remarkably, the Reisman Group was able to synthesize each fragment from a common starting material, using the same general strategy as for C-C bond formation and spiroketalization. Thus, steroids **33** and **34** can be traced back retrosynthetically to the corresponding alkynes **35** and **36**, where the gold-mediated cycloisomerization would be used to form the respective spiroketals. It was envisioned that alkynes **35** and **36** could be obtained by the 1,2-addition of the propargyl metal species derived from the appropriate alkynes to the alpha-hydroxy ketone accessible on *trans*-dehydroandrosterone (**37**).

**Scheme 2:**
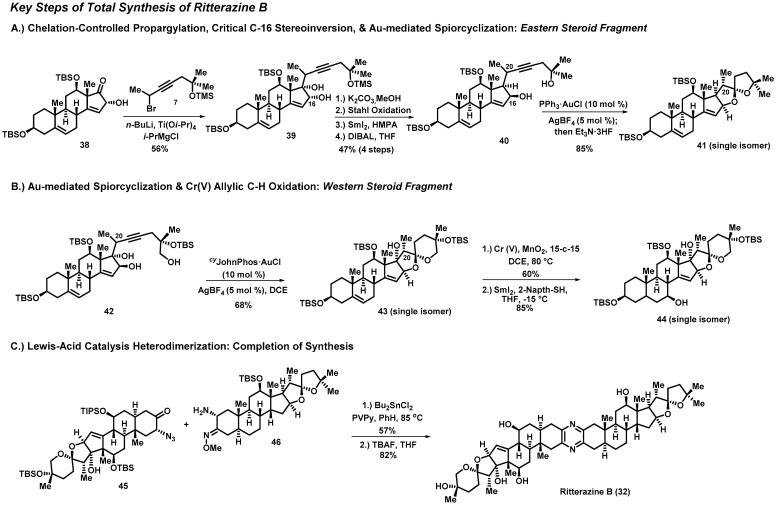
Key steps in Reisman’s total synthesis of ritterazine B (**32**).

The first key steps in the total synthesis of ritterazine B are a set of divergent titanium-mediated propargylations, which provide two different alkynes that serve as starting materials for each fragment. For the eastern fragment, treatment of **38** with *n*-butyl lithium results in deprotonation of the C16 alcohol, followed by 1,2-addition of the organotitanium species derived from propargyl bromide, yielding the alkyne **39** (Scheme 2). It is important to note that these additions for both fragments occur with exclusive beta-face selectivity, despite the neighboring axial methyl group. This is presumably due to the formation of an alpha-cyclic chelate between the C16 and C17 oxygens. Therefore, the alpha configuration at C16 is critical in imparting the desired stereocontrol in the divergent step. Additionally, this same C16 alcohol controls the stereochemical outcome of the gold-mediated spirocyclization, in which the beta configuration at C16 is needed in order to construct the required spiroketals. Critical stereoinversion of C16 can be achieved through a Stahl oxidation and a diastereoselective 1,2-reduction using di-*iso*-butylaluminum hydride to yield **40,** where the alcohol at C16 is now in the vital beta configuration.

The two-step stereoinversion sequence sets the stage for a gold-mediated spiroketalization—another key transformation in the synthesis of both the eastern and western fragments. Extensive experimentation revealed that treatment of the alkyne precursor (**40** or **42**) with *^Cy^*JohnPhos∙AuCl (10 mol%) and AgBF_4_ (5 mol%) provided the spiroketal (**41** or **43**) as a single diastereomer. It is important to note that this critical reaction not only provided the desired configuration at the spiroketals, but also resulted in the convergence of C20 epimers, delivering the spiroketals with the necessary alpha-disposed methyl group. An important late-stage transformation on the western steroid fragment is the allylic C-H oxidation of **43** using oxochromate (Cr(V)) and MnO_2_ as a co-oxidant. This rare chromium oxidation chemistry resulted in the enone intermediate (not shown), which was reduced using SmI_2_ to yield **44** as a single diastereomer.

In conclusion, the Reisman Group reported the first total synthesis of ritterazine B from a simple steroid starting material over 37 steps in a 9% overall yield. The synthesis featured a unique strategy, which included a divergent titanium-mediated propargylation from a common building block to construct the eastern and western fragments. A gold-catalyzed, diastereoselective spirocyclization elaborated each eastern and western steroid into their respective spiroketals (eastern fragment: 59% over 18 steps; western fragment: 33% over 17 steps). Lastly, a convergent tin-catalyzed heterodimerization combined **45** and **46** via an initial imine formation, subsequent loss of molecular nitrogen from the intermediate azide, and a final cyclization/aromatization sequence to provide the desired pyrazine moiety of ritterazine B, followed by global silyl deprotection using tetrabutylammonium fluoride (TBAF) to yield **32**. The Reisman Group has employed their route to generate several multimilligram batches of ritterazine B, and investigations into the biological activity of **32** are currently underway.

### 3.2. Total Synthesis of Cephalostatin 1

**Scheme 3:**
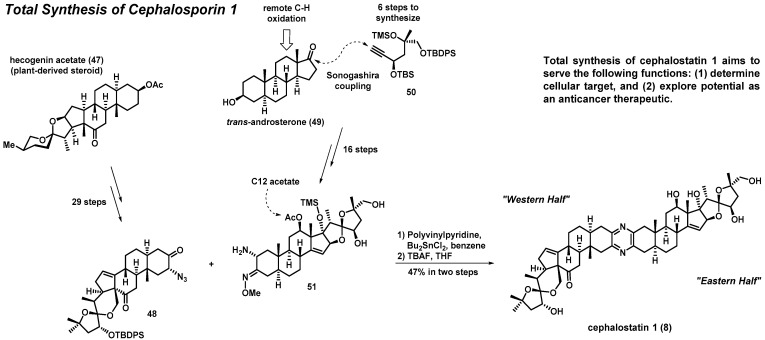
Overview of Shair’s total synthesis of cephalostatin 1 (**8**).

Shair et al. have reported a convergent enantioselective synthesis of cephalostatin 1 (Scheme 3) [45]. The total synthesis approach to cephalostatin 1 hinged on a late-stage heterodimerization of key “western” and “eastern” fragments through a pyrazine-forming reaction to condense distinct α-aminoketone synthons. The “western half” fragment of cephalostatin 1 was synthesized in 29 steps from the plant-derived steroid hecogenin acetate (**47**) (available at kilogram scale). The synthesis of the “eastern half” of cephalostatin 1 was carried out in 16 steps from *trans*-androsterone (**49**), and included (1) a remote C-H oxidation (C-ring) and (2) installation of an alkyne side chain via Sonogashira coupling with **50**. The critical late-stage pyrazine-forming reaction occurred between α-azidoketone **48** and α-aminomethyloxime **51** upon treatment with polyvinylpyridine and dibutyltin dichloride (Bu_2_SnCl_2_). Final treatment with TBAF removed silyl- and acetate-protecting groups to produce cephalostatin 1 (**8**) at a 47% yield over the final two steps.

### 3.3. Total Syntheses of (-)-Barrenazines A and B

**Scheme 4:**
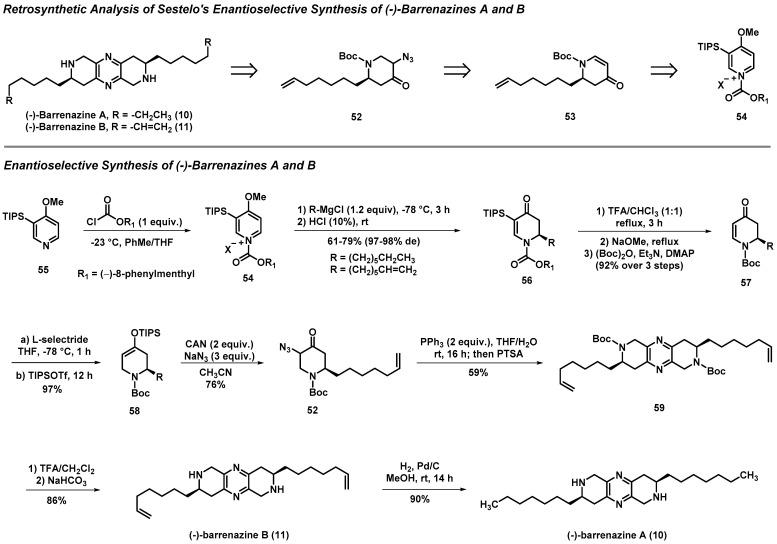
Sestelo’s retrosynthetic plan and asymmetric synthesis of (-)-barrenazines A (**10**) and B (**11**).

Barrenazines A and B are structurally interesting pyrazine natural products isolated from a tunicate in the Barren Islands, and demonstrate anticancer activities against colon carcinoma, prostate carcinoma, and leukemia cells. Due to their interesting molecular structures and biological activities, barrenazines A and B have generated considerable interest from the synthetic community. Sestelo reported enantioselective total syntheses of (-)-barrenazines A and B using an (-)-8-phenylmenthyl carbamate—initially developed by Comins—as a chiral auxiliary to direct Grignard additions into an *N*-acylpyridinium salt (**54**) and establish the absolute stereochemistry in these natural products (Scheme 4) [46]. Following the key Grignard reaction to yield **56** with high enantioselectivity, enone **57** was subjected to L-selectride (conjugate) reduction and subsequent treatment with TIPSOTf to trap the intermediate enolate species in order to form silyl enol ether **58** at a 97% yield. Treatment of **58** with ceric ammonium nitrate (CAN) and sodium azide afforded α-azidoketone **52** at a 76% yield. Staudinger reduction of **52** afforded the corresponding α-aminoketone (structure not shown), which was then treated with PTSA to generate pyrazine **59** at a 59% yield. Boc group deprotection of **59** was carried out following treatment with trifluoroacetic acid (TFA) to afford (-)-barrenazine B (**11**) at an 86% yield. Finally, **11** was hydrogenated to produce (-)-barrenazine A (**10**) at a 90% yield.

### 3.4. Total Synthesis of Dragmacidin D through Advances in C-H/C-H Cross-Coupling

**Scheme 5:**
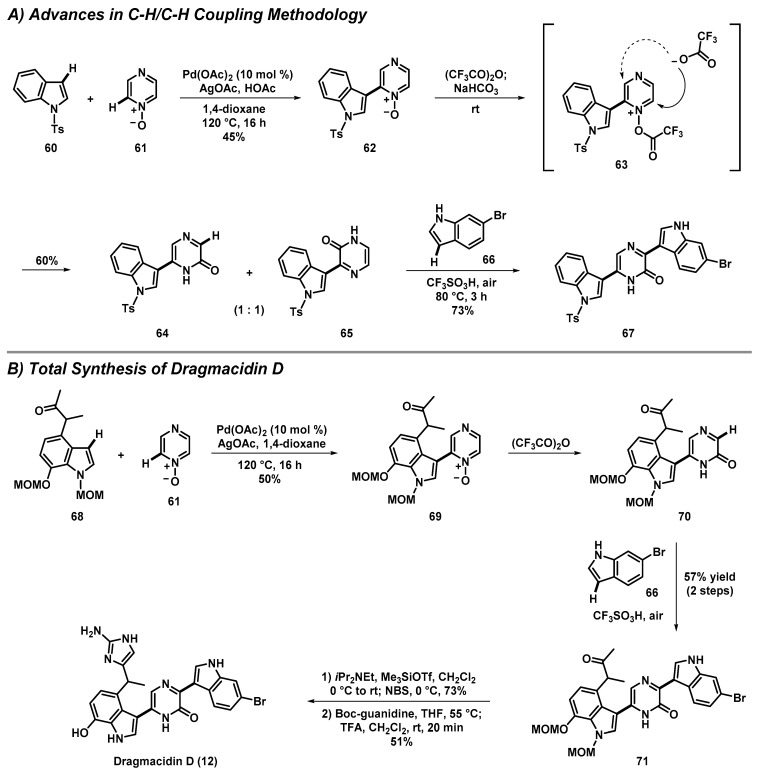
Itami and Yamaguchi’s total synthesis of dragmacidin D (**12**) using an innovative C-H/C-H coupling methodology.

Dragmacidin D is a marine natural product that demonstrates potent inhibition of serine/threonine protein phosphatases, which have implications for the treatment of Alzheimer’s, Parkinson’s, and Huntington’s diseases. Itami, Yamaguchi, et al. disclosed an interesting synthetic approach to dragmacidin D model compound **67,** involving a series of innovative C-H/C-H cross-coupling reactions (Scheme 5) [47]. The initial C-H/C-H cross-coupling between indole **60** and mono *N*-oxide of pyrazine (**61**) was catalyzed by palladium(II) acetate and silver(I) acetate to afford **62** at a 45% yield. Following this, *N*-oxide **62** was subjected to trifluoroacetic anhydride to give a 1:1 mixture of pyrazinones **64** and **65** at a 60% yield. 5-Indolopyrazinone **64** was then subjected to a second C-H/C-H cross-coupling with 6-bromoindole (**66**) and trifluoromethanesulfonic acid (CF_3_SO_3_H) in DMF, in the presence of air, to form **67** at a 73% yield. This model synthesis laid the foundation for a concise total synthesis of dragmacidin D.

The total synthesis of dragmacidin D was carried out in only five steps, starting from MOM-protected indole **68**, which was accessed in nine steps. Compound **68** was subjected to a palladium(II)-acetate-catalyzed C-H/C-H cross-coupling reaction with the pyrazine-*N*-oxide (**61**) to afford **69** at a 50% yield [47]. Compound **69** was then treated with trifluoroacetic anhydride and successfully converted to pyrazinone **70,** before a second C-H/C-H cross-coupling reaction was successfully carried out with 6-bromoindole to yield **71** (57% yield over two steps; Scheme 5). The end-game synthetic sequence included (1) α-bromination of the ketone in **71** following silyl enol formation and subsequent treatment with NBS (73% yield), (2) installation of Boc-protected 2-aminoimidazole from treating the α-bromoketone intermediate with Boc-guanidine, and (3) a final reaction with trifluoroacetic acid to promote global deprotection and access (±)-dragmacidin D (**12**) (51% yield over two steps). This unique synthetic approach showcases multiple C-H/C-H cross-coupling reactions, which enable rapid access to this important marine alkaloid. In addition, this total synthesis of dragmacidin D provides a clear roadmap towards accessing related alkaloids and novel synthetic analogues for biological investigations relevant to various neurological diseases.

### 3.5. Total Synthesis of Botryllazine Involving a New C-H Functionalization of Pyrazines

**Scheme 6:**
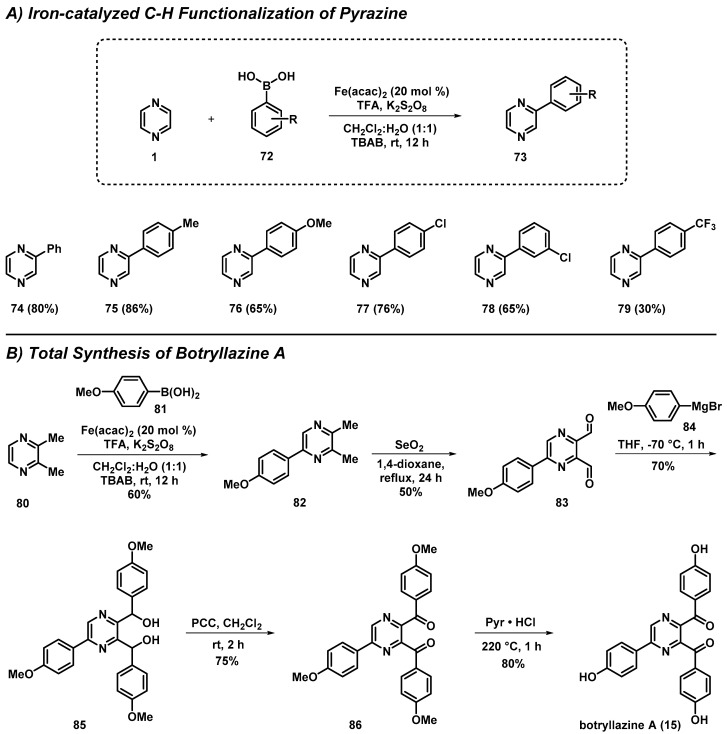
Singh’s synthesis of botryllazine A (**15**) utilizing an iron-catalyzed C-H coupling method of pyrazines.

Singh et al. developed an iron-catalyzed C-H functionalization of electron-deficient heterocycles with organoboron agents. In addition, this robust methodology was used to complete a short total synthesis of the natural product botryllazine A (**15**) (Scheme 6) [48]. The work aimed to address challenges related to the cross-coupling of electron-poor heteroarenes, including pyrazines. During the course of these studies, arylboronic acids (**72**) were successfully coupled to pyrazines **1** and **80**, with up to 86% yield, using a combination of 20 mol % iron(II) acetylacetonate [Fe(acac)_2_], one equivalent of trifluoroacetic acid, a phase-transfer catalyst (tetrabutylammonium bromide; TBAB) and an oxidant (potassium persulfate; K_2_S_2_O_8_) in 1:1 dichloromethane:water. This new method transformed 2,3-dimethylpyrazine (**80**) to arylated pyrazine **82** (60% yield), which was further elaborated to botryllazine A (**15**) using a short synthetic sequence involving benzylic oxidation to dialdehyde **83**, Grignard addition with **84** to yield intermediate **85**, subsequent PCC oxidation to diketone **86** and, finally, acid-promoted demethylations to botryllazine A (**15**).

### 3.6. Total Synthesis of the Pyrazine Bisindole Alkaloid Alocasin A

**Scheme 7:**
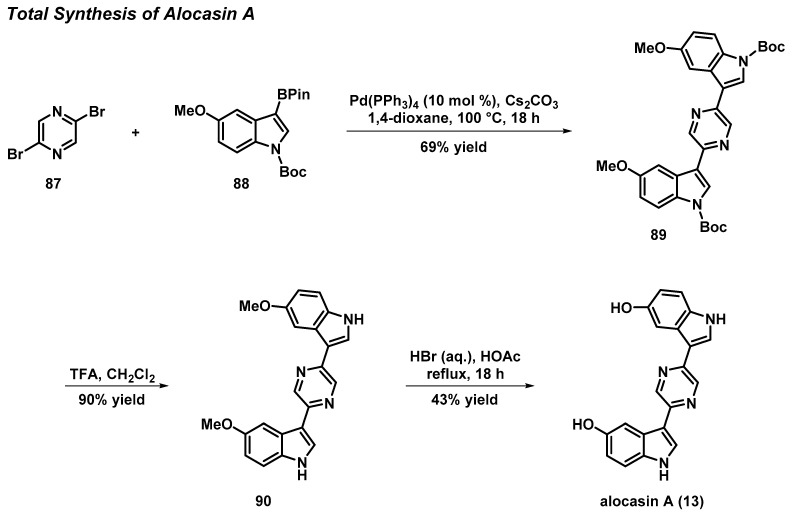
Sperry and Kim’s total synthesis of alocasin A (**13**).

Alocasin A is a pyrazine-linked bisindole alkaloid isolated from the plant *Alocasia macrorrhiza* and used throughout southern Asia as folk medicine to treat headaches, flu, and other conditions. Sperry and Kim developed a concise total synthesis of alocasin A that was initiated with a double Suzuki–Miyaura coupling between 2,5-dibromopyrazine (**87**) and 3-borylindole **88** using tetrakis(triphenylphosphine)palladium(0) to afford pyrazine bisindole **89** at a 69% yield (Scheme 7) [49]. The final sequence included an *N*-Boc-group removal upon treatment with trifluoroacetic acid (90% yield), followed by a final demethylation of **90** with hydrobromic acid to yield alocasin A (**13**) (43% yield). This robust synthetic route enables rapid access to alocasin A, while providing a roadmap to generate structurally related synthetic analogues for more extensive biological studies.

### 3.7. Total Synthesis of Wasp Pheromone 2-Hydroxymethyl-3-(3-methylbutyl)-5-methylpyrazine

**Scheme 8:**
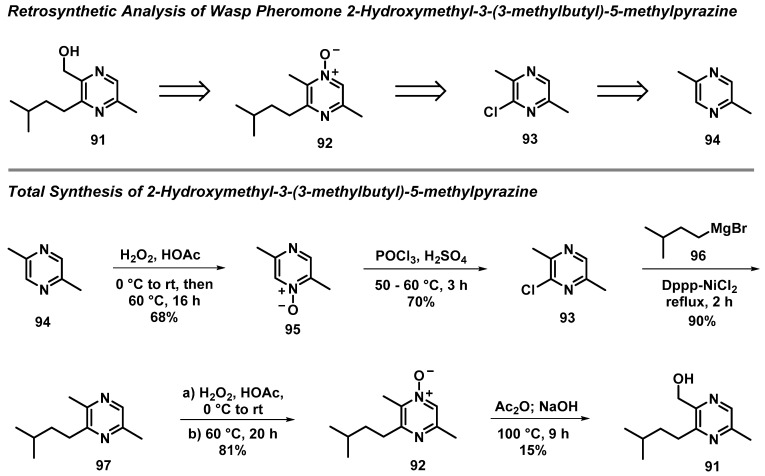
Barrow’s synthesis of 2-hydroxymethyl-3-(3-methylbutyl)-5-methylpyrazine (**91**) via the use of *N*-oxide intermediates and a nickel-catalyzed Kumada–Corriu cross-coupling reaction.

An elegant synthesis of the female wasp pheromone 2-hydroxymethyl-3-(3-methylbutyl)-5-methylpyrazine (**91**) was reported by Barrow et al. using a five-step synthetic sequence [50]. This instructive route demonstrates the incredible synthetic utility of *N*-oxides in the synthesis of pyrazine-based molecules. In this synthesis, *N*-oxide intermediates were readily accessed to strategically install a chlorine atom at the 2 position of the pyrazine heterocycle and facilitate a Boekelheide rearrangement in the last step to install the hydroxymethyl group of **91**. In addition, 2-chloropyrazine **93** was an ideal substrate for a nickel-catalyzed Kumada–Corriu cross-coupling with 3-methylbutylmagnesium bromide (**96**) to afford trisubstituted pyrazine **97** at a 90% yield (Scheme 8). The robust strategies employed in this streamlined synthesis are highly practical, and could be applied to access numerous pyrazine compounds in the future.

### 3.8. Regio- and Chemoselective Metallations to Access the Pyrazine Natural Product Coeleterazine

**Scheme 9:**
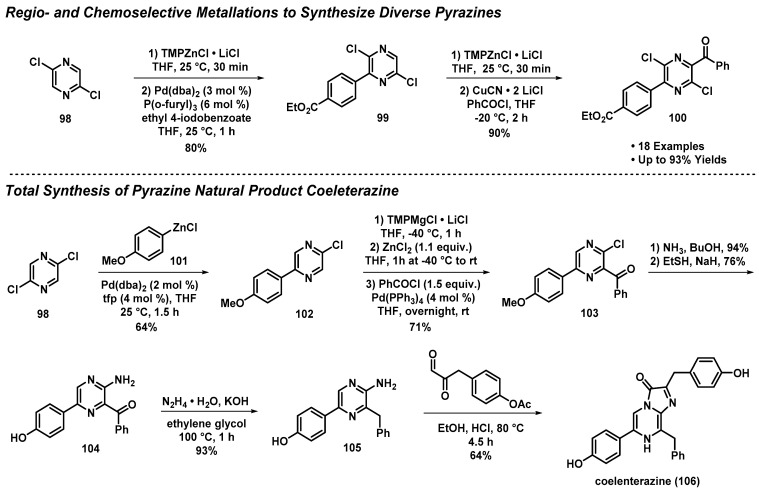
Knochel’s total synthesis of coelenterazine (**106**) by use of regio- and chemoselective metalation reactions.

Knochel et al. developed a sequential regio- and chemoselective metalation methodology by treating chloropyrazine **98** with TMPMgCl·LiCl (2,2,6,6-tetramethylpiperidide magnesium chloride; Turbo-Hauser base) and (2,2,6,6-tetramethylpiperidide magnesium chloride; Turbo-Hauser base) and TMPZnCl·LiCl. Subsequent trapping of metallated intermediates was effective following treatment with various electrophiles/cross-coupling partners (e.g., aryl iodide), and afforded highly functionalized pyrazine compounds (e.g., **100**; Scheme 9) [51]. This methodology was demonstrated to provide 18 pyrazines, showcasing the synthetic utility of this high-yielding sequence (≤93% yields), which enables considerable diversification of the heterocyclic nucleus. This robust synthetic methodology was used to synthesize coelenterazine (**106**)—a naturally occurring bioluminescent pyrazine produced by the jellyfish *Aequorea victoria*—in eight linear steps from **98** (19% overall yield).

### 3.9. Total Synthesis of Pyrazine Alkaloids from Amino Acids

**Scheme 10:**
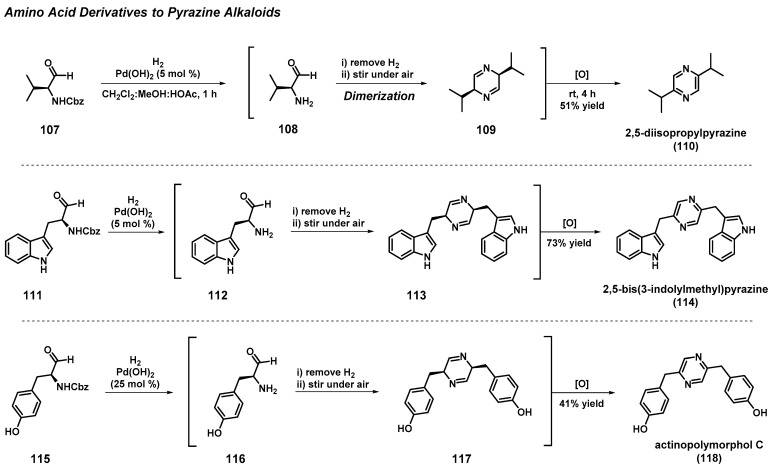
Biomimetically inspired synthesis of 2,5-disubstituted pyrazine alkaloids.

A biomimetically inspired synthesis of 2,5-disubstituted pyrazine alkaloids was accomplished through the homodimerization of α-amino aldehydes and subsequent air oxidation (Scheme 10) [52]. Cbz-protected α-amino aldehydes (**107**, **111**, **115**) that were required for these total syntheses efforts were readily synthesized from common amino acids. Each α-amino aldehyde reported in this study was accessed from hydrogenolysis of the Cbz-protecting group upon treatment with 5 mol % palladium(II) hydroxide under a hydrogen atmosphere, followed by the critical tandem condensation–oxidation sequence to afford target pyrazine alkaloids at 41–73% yields (2,5-diisopropylpyrazine (**110**), 51% yield; 2,5-bis(3-indolylmethyl)pyrazine (**114**), 73% yield; actinopolymorphol C (**118**), 41% yield).

### 3.10. Total Synthesis of Favipiravir from 2-Aminopyrazine

**Scheme 11:**
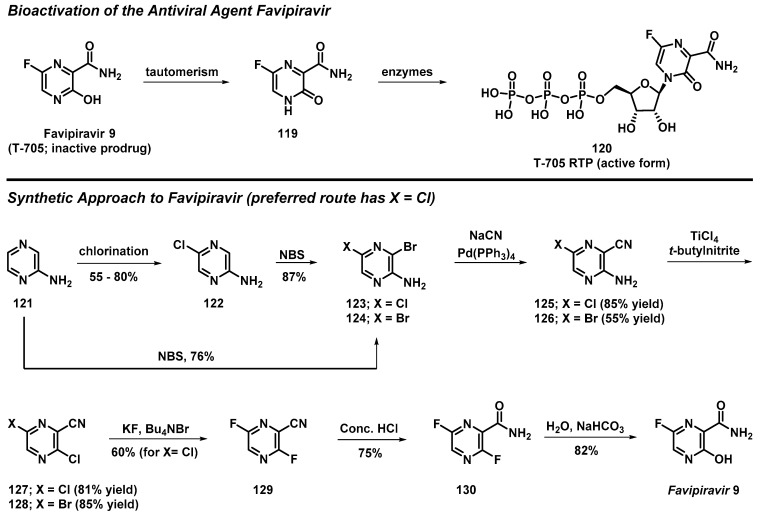
Guo’s chemical synthesis of the antiviral agent favipiravir (**9**).

Guo et al. recently reported the total synthesis of favipiravir (**9**, T-705)—a pyrazine-containing antiviral prodrug that requires enzyme action to produce its active favipiravir-ribofuranosyl-5′-triphosphate (**120**, RTP) form (Scheme 11) [16]. In this work, favipiravir was synthesized using three synthetic routes; however, the preferred route required seven steps from commercially available 2-aminopyrazine (**121**), and was highlighted by an efficient synthesis of 3,6-dichloropyrazine-2-carbonitrile (**127**). This key synthetic intermediate was prepared in four steps from 2-aminopyrazine, which included (1) a regioselective chlorination of the pyrazine heterocycle to **122**, (2) NBS bromination to **123**/**124**, (3) palladium-catalyzed cyanation to form **125**/**126**, and (4) a Sandmeyer diazotization/chlorination sequence to yield **127**/**128**. This synthetic approach eliminated the need for POCl_3_, and afforded a better yield (48%) than the alternative routes reported in this study (not detailed here). Intermediates **127**/**128** were subjected to a nucleophilic aromatic substitution reaction with fluorine, followed by partial hydrolysis of the nitrile moiety to form amide **130**; finally, nucleophilic aromatic substitution with water yielded favipiravir (**9**).

## 4. Recent Total Synthesis of Phenazine-Containing Natural Products

### 4.1. Total Synthesis of N-Alkyl-2-halophenazin-1-ones

**Scheme 12:**
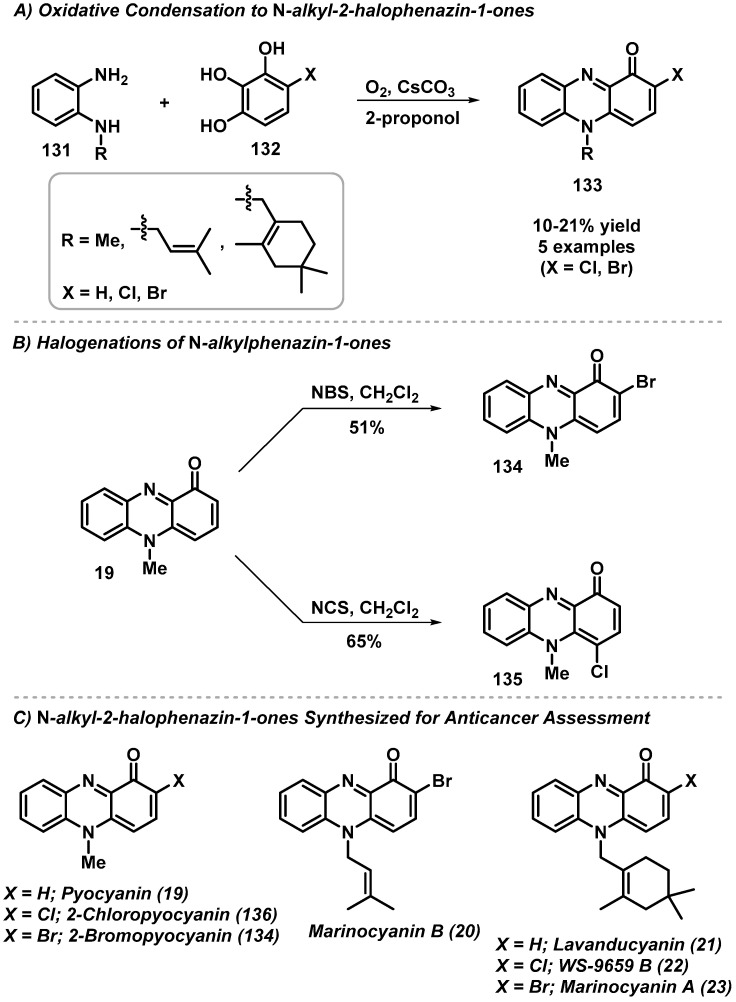
Kuromachi’s total synthesis of *N*-alkyl-2-halophenazin-1-one natural products.

Several natural *N*-alkyl-2-halophenazin-1-one products are of considerable interest due to their biological activity profiles, including pyocyanin (**19**), marinocyanin B (**20**), lavanducyanin (**21**), WS-9659 B (**22**), and marinocyanin A (**23**). Kuromachi et al. recently reported the synthesis of six naturally occurring *N*-alkyl-2-halophenazin-1-ones using an oxidative condensation of *N*-alkylbenzene-1,2-diamines (**131**) with 4-halo-1,2,3-benzenetriol (**132**) (5 examples, 10–21% yield; Scheme 12) [53]. This new synthetic approach enables the synthesis of *N*-alkyl-2-chlorophenazin-1-ones, whereas standard *N*-chlorosuccinimide chlorination of *N*-alkyl-phenazin-1-ones provides an undesired chlorination at the 4 position of the phenazine heterocycle.

Following chemical synthesis, cytotoxicity of six *N*-alkyl-2-halophenazin-1-ones and three *N*-alkylphenazin-1-ones against human promyelocytic leukemia HL-60, lung cancer A549, and non-cancerous MRC-5 cells was investigated. Results from the biological assessment show lavanducyanin (**21**) and the 2-position-halogenated analogues demonstrating the most potent cytotoxicity (IC_50_ ≤ 0.75 µM), while marinocyanin B (**20**) analogues show good cytotoxicity (IC_50_ = 0.33–2.59 µM); however, these compounds were also found to be cytotoxic to the same degree as the non-cancerous cell lines (MRC-5). Interestingly, 2-chloropyocyanin (**136**) possessed the most ideal selectivity profile against A549 cells (IC_50_ = 0.76 µM) when compared to MRC-5 (IC_50_ = 4.41 µM).

### 4.2. Total Synthesis and Antileukemic Evaluation of Iodinin, Myxin and Derivatives

**Scheme 13:**
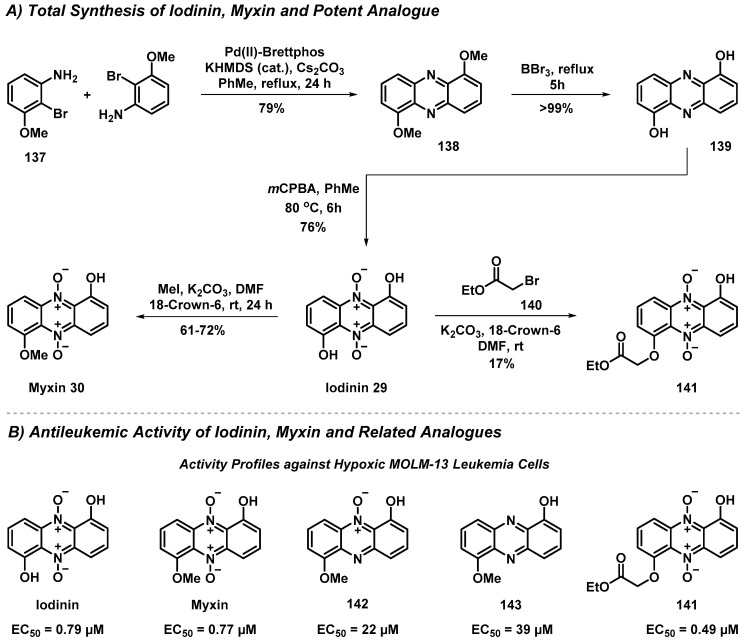
Rongved’s total synthesis of iodinin (**29**), myxin (**30**), and related analogues for evaluation against leukemia cancer cells.

An efficient total synthesis of the naturally occurring phenazine 5,10-dioxides iodinin (**29**) and myxin (**30**) was recently reported by Rongved et al. (Scheme 13) [54]. In addition, a series of related synthetic analogues was generated (e.g., **141**–**143**), and their activity against leukemia cells was investigated to determine cytotoxicity in hypoxic and non-hypoxic conditions. This work provided considerable insights into the structure–activity relationship profiles of these interesting anticancer agents, while paving the way for future developments in this area.

The synthesis of iodinin was initiated with a double palladium-catalyzed Buchwald–Hartwig C-N cross-coupling of 2-bromo-3-methoxyaniline (**137**) to generate 1,6-dimethoxyphenazine (**138**) at a 79% yield on a one-gram scale. Numerous palladium catalyst systems were screened to carry out the 1,6-dimethoxyphenazine synthesis; however, the best results were obtained using the palladium(II)-BrettPhos precatalyst in combination with potassium hexamethyldisilazide (KHMDS). Next, 1,6-dimethoxyphenazine was demethylated to afford 1,6-dihydroxylphenazine (**139**) using boron tribromide (BBr_3_) at a near-quantitative yield, followed by double *N*-oxidation using *m*CPBA in toluene to afford iodinin (**29**) at a 76% yield. This robust synthetic route afforded iodinin at a gram scale, which enabled rapid access to myxin (**30**) at a 61–72% yield following treatment with methyl iodide and potassium carbonate in DMF with the addition of 18-Crown-6. In addition, iodinin was alkylated with α-bromo esters (e.g., **140**) to further explore synthetic analogues related to myxin.

Following chemical synthesis, iodinin, myxin, and a diverse series of related analogues were evaluated against MOLM-13 leukemia cells in hypoxic (low, 2% oxygen concentration) and normoxic (normal, 19% oxygen concentration) conditions. Agents that selectively target hypoxic cancer cells are of considerable interest, as cancers that thrive in low-oxygen environments are notoriously aggressive and lead to poor prognoses in patients. Results from these *in vitro* experiments demonstrated that iodinin, myxin, and multiple structurally related analogues (e.g., **141**) showed potent and increased activity against hypoxic MOLM-13 leukemia cells (e.g., iodinin: EC_50_ = 2.0 µM against normoxic cells, EC_50_ = 0.79 µM against hypoxic cells). In addition, analogues **142** and **143** were designed to probe the necessity for the double *N*-oxide feature in iodinin/myxin, and showed significant losses in activity against MOLM-13 cells. This was the first evidence that iodinin (**29**) demonstrated hypoxia-selective antileukemia activity, and has the potential to act on malignant cells in hypoxic bone marrow of AML patients.

### 4.3. Total Synthesis of Streptophenazine A and G

**Scheme 14:**
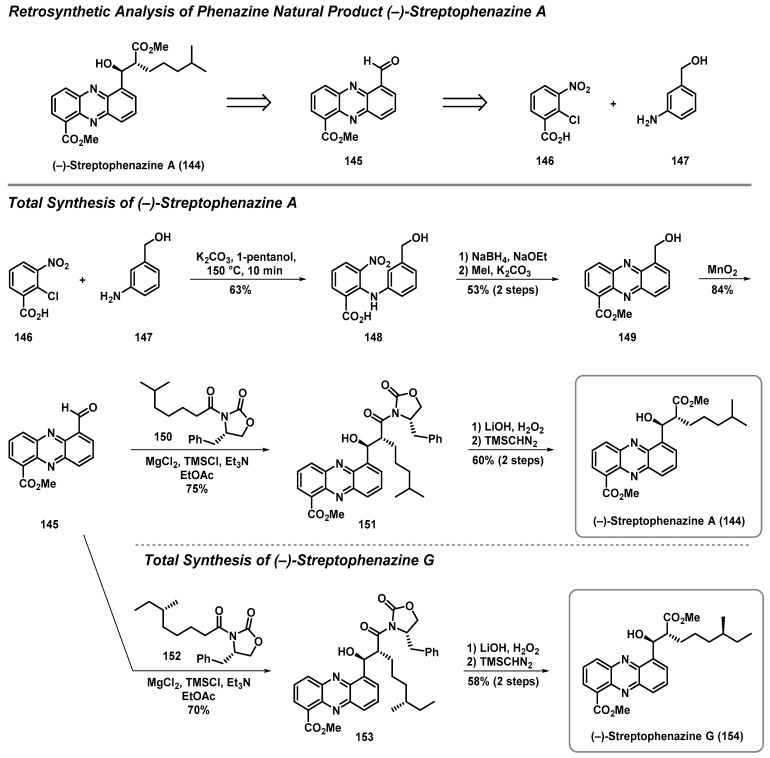
Yang’s asymmetric synthesis of (-)-streptophenazines A (**144**) and G (**154**).

Streptophenazines A–H are a structurally related series of natural phenazine products isolated from marine *Streptomyces* sp. strain HB202. Streptophenazine A was isolated in 2008, and reported moderate antibacterial activity, which garnered interest from the scientific community. Yang et al. completed the first asymmetric synthesis of (-)-streptophenazine A (**144**), which proved critical to its structural elucidation, as this chemical synthesis led to a revision of the initially reported structure (Scheme 14) [55]. In addition, this synthesis allowed for accurate stereochemical assignment regarding the two stereogenic centers of (-)-streptophenazine A.

In terms of the retrosynthetic analysis, the plan to access (-)-streptophenazine A (**144**) hinged on a late-stage asymmetric aldol reaction from the key phenazine aldehyde **145** (Scheme 14). A disconnection through the central ring of the phenazine nucleus of **145** led Yang et al. to nitroarene **146** and aniline **147** as building blocks to start the synthesis. This robust synthesis plan proved to be quite flexible, and was also utilized to synthesize (-)-streptophenazine G (**154**) [56].

The phenazine heterocycle of (-)-streptophenazine A (**144**) was rapidly assembled using a short synthetic sequence that included a nucleophilic aromatic substitution (S_N_Ar) reaction between **146** and **147**, followed by a reductive cyclization reaction with sodium borohydride/sodium ethoxide of **148** to form the critical phenazine heterocycle (Scheme 14) [55]. Next, treatment with methyl iodide and potassium carbonate resulted in the formation of methyl ester **149**. Subsequent benzylic oxidation with manganese oxide yielded aldehyde **145** as a key intermediate to this modular synthetic platform. To complete the synthesis of (-)-streptophenazine A, aldehyde **145** was subjected to an asymmetric aldol reaction with the enolate of **150** to set the two stereocenters of **151** with high levels of stereochemical control at a 75% yield. The end-game sequence to (-)-streptophenazine A involved the cleavage of the oxazolidinone moiety of **151** with lithium hydroxide and hydrogen peroxide, and final installation of the methyl ester upon treatment of the corresponding carboxylic acid (structure not shown) with (trimethylsilyl)diazomethane (TMSN_2_) to yield (-)-streptophenazine A (**144**).

In addition, Yang reported an analogous synthesis to (-)-streptophenazine G (**154**) (Scheme 14) [56]. Similar to the (-)-streptophenazine A study, the chemical synthesis of (-)-streptophenazine G was critical to determining the absolute stereochemistry of this natural product, which possesses a hydrocarbon side chain bearing an additional stereocenter when compared to (-)-streptophenazine A. During these investigations, the key aldehyde **145** was subjected to an asymmetric aldol reaction with oxazolidinone **152** bearing the appropriate side chain. From **153**, a hydrolysis/methyl ester formation end-game sequence yielded (-)-streptophenazine G (**154**). Collectively, the modular synthesis that Yang et al. established to rapidly access streptophenazine natural products and derivatives could make a significant impact towards the treatment of antibiotic-resistant bacterial pathogens.

### 4.4. Marine Phenazine 2-Bromo-1-Hydroxyphenazine-Inspired Antibacterial Agents

Our group has discovered a series of synthetically tunable halogenated phenazine antibacterial agents that eradicate surface-attached biofilms with excellent potency [24,25,57,58,59,60,61]. Biofilms are densely packed bacterial communities that consist of enriched populations of metabolically-dormant (non-replicating) persister cells demonstrating high levels of tolerance to all classes of antibiotics. Biofilms are highly prevalent, and are credited as the underlying cause of chronic and recurring infections in humans.

Our program was initially inspired by the action of phenazine antibiotics in the lungs of cystic fibrosis (CF) patients. When CF patients are young, they often endure chronic lung infections initially caused by the Gram-positive bacterial pathogen *Staphylococcus aureus*. As these CF patients age, *Pseudomonas aeruginosa* co-infects the lungs and eradicates *S. aureus* through the use of redox-active phenazine antibiotics. From this interesting competitive interaction between *P. aeruginosa* and *S. aureus*, we hypothesized that phenazine heterocycles would be a good starting point to discover novel antibacterial agents that could potentially eradicate established biofilms [58].

**Scheme 15:**
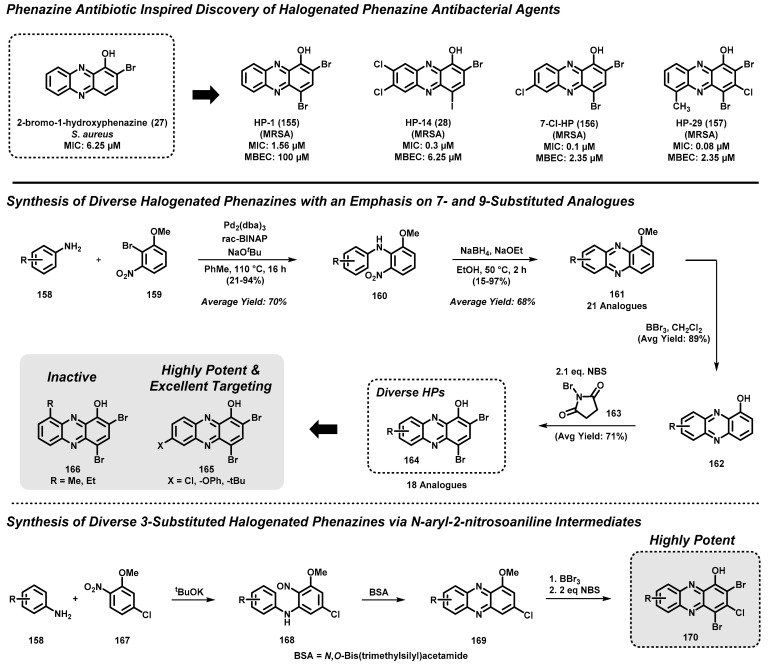
Huigens lab’s key synthesis efforts related to halogenated phenazines that demonstrate potent antibacterial and biofilm-eradicating activities.

During initial investigations, we synthesized a panel of five phenazine antibiotics (e.g., pyocyanin) and eight diverse phenazine derivatives of synthetic origin. In the initial MIC assay against planktonic *S. aureus* bacteria, we found 2-bromo-1-hydroxyphenazine (**27**) to be the most potent natural phenazine product in our focused panel, with an MIC = 6.25 µM (Scheme 15), which was significantly more potent than that of pyocyanin (**19**; MIC = 50 µM) [57]. In addition, we found the related synthetic compound 2,4-dibromo-1-hydroxyphenazine **155** (halogenated phenazine analogue 1, or **HP-1**) to demonstrate the most potent antibacterial activity during initial studies, with an MIC = 1.56 µM against *S. aureus*. In follow-up studies, we showed that **HP-1** was able to eradicate established methicillin-resistant *S. aureus* biofilms, with a minimum biofilm eradication concentration (MBEC) of 100 µM [58,59].

After the identification of **HP-1**, we wanted to explore structure–activity relationships (SARs) of the HP scaffold by functionalizing the other positions on the phenazine heterocycle with various substituents (Scheme 15) [24,58,59,60,61]. To do this, we devised modular chemical syntheses that enabled a diversity of functionalization at the 3- and 6-9 positions of the HP scaffold from available aniline starting materials. Precursor 1-methoxyphenazines (e.g., **161**, **169**) were rapidly accessed through a number of efficient synthetic routes, including (1) Buchwald–Hartwig cross-coupling reaction between diverse anilines **158** and 2-bromo-3-nitroanisole **159**, followed by a sodium-borohydride-mediated reductive cyclization to form the phenazine nucleus of **161**; [24] and (2) a potassium-*tert*-butoxide-promoted reaction between aniline starting materials (**158**) and 5-chloro-2-nitroanisole **167** to form *N*-aryl-2-nitrosoaniline intermediates **168**, which were then subjected to *N*,*O*-Bis(trimethylsilyl)acetamide (BSA) to form the desired phenazine heterocycle [61]. From these synthetic efforts, 1-methoxyphenazine products (e.g., **161** and **169**) were then demethylated using boron tribromide (BBr_3_), and brominated with two equivalents of *N*-bromosuccinimide **163** (NBS) to yield target halogenated phenazines **164** and **170** [24,61].

Overall, this collection of novel halogenated phenazines (e.g., **28**, **155**–**157**) demonstrate potent antibacterial activities against several Gram-positive pathogens, including *Staphylococcus aureus*, *Staphylococcus epidermidis*, and *Enterococcus faecalis* (antibacterial activities reported as minimum inhibitory concentrations, or MIC values) [24,25,57,58,59,60,61]. In addition, halogenated phenazines potently eradicate surface-attached bacterial biofilms that are innately tolerant to all classes of conventional antibiotics (biofilm-killing activities reported as minimum biofilm eradication concentrations, or MBEC values). HP-14 (**28**) is one of the potent analogues we discovered during the course of these studies, and our group used this compound as a probe in transcriptomic profiling experiments to demonstrate that HPs induce rapid iron starvation in MRSA-1707 biofilms [25]. We have shown that several other HP analogues—such as **HP-29** (**157**)—also induce rapid iron starvation in MRSA biofilms [61]. In addition, **HP-29** has demonstrated good efficacy in treating *S. aureus* and *Enterococcus faecalis* wound infections in mice. Collectively, small HP molecules have the potential to dramatically impact future therapies related to biofilm-associated infections in humans.

## 5. Conclusions

Several exciting studies and discoveries regarding pyrazine- and phenazine-containing compounds have been reported in recent years. This review covers a diverse collection of outstanding contributions regarding total syntheses and medicinal chemistry efforts related to pyrazine- and phenazine-containing compounds. In addition, we discussed many pyrazine and phenazine agents that have shown potential therapeutic value, including several that are clinically used to treat disease in humans. Overall, pyrazine and phenazine compounds have produced significant interest from the medicinal and synthetic communities, and we anticipate continued advances regarding these exciting heterocycles.

## Dedication

We would like to dedicate this review to Professor Daniel Comins for his incredible contributions to heterocyclic chemistry, total synthesis, and medicinal chemistry. Not only did Dr. Comins pioneer his pyridine-based triflating reagent, but he also led incredible total synthesis efforts related to complex natural alkaloid products. In addition, his efforts significantly advanced our understanding of camptothecin anticancer agents and nicotine derivatives. Dr. Comins led a talented research lab of graduate and postdoctoral students who have gone on to do incredible work in their independent careers. Finally, Dr. Comins has been an outstanding educator to a generation of graduate students at North Carolina State University through developing courses for advanced synthesis and heterocyclic chemistry that have had a lasting impact on many, adding to his legacy in ways that cannot be measured.
